# Epidemiology of foodborne disease outbreaks from 2011 to 2016 in Shandong Province, China

**DOI:** 10.1097/MD.0000000000013142

**Published:** 2018-11-09

**Authors:** Guangjian Wu, Qun Yuan, Liansen Wang, Jinshan Zhao, Zunhua Chu, Maoqiang Zhuang, Yingxiu Zhang, Kebo Wang, Peirui Xiao, Ya Liu, Zhongjun Du

**Affiliations:** a Shandong Center for Disease Control and Prevention; b Shandong Center for Food Safety Risk Assessment; c Academy of Preventive Medicine, Shandong University, Jinan, Shandong Province; d School of Public Health, Jilin University, Changchun, Jilin Province; e Shandong Academy of Occupational Health and Occupational Medicine, Shandong Academy of Medical Sciences, Jinan, Shandong Province, People's Republic of China.

**Keywords:** epidemiology, foodborne diseases, outbreaks, public health, surveillance

## Abstract

Foodborne disease is a major public health concern in both developed and developing countries. China has established a nationwide Foodborne Disease Outbreak Surveillance System (FDOSS) for collection and periodic reporting of data on the occurrence and causes of foodborne disease outbreaks in China. Each provincial Centers for Disease Control and Prevention (CDC) conducts the system working.

We reviewed foodborne disease outbreaks that occurred during 2011 to 2016 in Shandong Province from the FDOSS. The Wilcoxon test was used to compare the median number of ill persons in outbreaks. All data analysis was performed using Epi Info 7.

During 2011 to 2016, Shandong CDC received reports of 1043 foodborne disease outbreaks, resulting in 8078 illnesses, 2442 hospitalizations, and 17 deaths. There were a median of 69 outbreaks annually [interquartile range (IQR) 10–342], resulting in 335 to 3824 illnesses each year. The median outbreak size was 3 persons (IQR 2–7). Hotels (including cruise ships, hotpot restaurants, barbecue shops) were the most common setting. Among the 744 (71.3%) outbreaks with an implicated food or contaminated ingredient reported, 704 (94.6%) could be assigned to one of 17 predefined commodity categories. Of the 280 outbreaks with a known etiology, 117 (41.8%) were caused by poisonous plants and animals and their toxins, 39 (13.9) were caused by nitrite, and 27 (9.6%) were caused by vibrio parahaemolyticus. Of the 491 (47.1%) outbreaks with at least a contributing factor to cause outbreak, 168 (34.2%) were caused by improper processing, and 100 (20.4) were caused by inedible and misuse.

Timely investigation, disposal and reporting of foodborne disease outbreaks provides information that might help FDOSS to make full use of efficiency and FDOSS should be continued and strengthened even more in Shandong Province, such as an increase in diagnostic laboratory capacities.

## 1 Introduction

Foodborne diseases comprise a broad spectrum of diseases and account for a significant proportion morbidities and mortalities worldwide, it is a major public health concern in both developed and developing countries.^[1]^ The exact mortality associated with foodborne illnesses is difficult to determined.^[2]^ However, gastrointestinal illness caused about 2 million deaths worldwide during the year 2005.^[3]^ Various pathogens or toxins can cause more than 250 different foodborne illnesses.^[4]^ Even though the causative agents of most cases of foodborne diseases are unknown, bacteria and viruses are the most likely causative agents from the worldwide.^[5,6]^ An estimated 9.4 million illnesses, 56,000 hospitalizations, and over 1300 deaths caused by a known pathogen occur every year in the United States.^[7]^

Foodborne diseases results from the consumption of food contaminated with pathogens such as bacteria, viruses, parasites or with poisonous chemicals or bio-toxins.^[8,9]^ Majority of the foodborne illness cases are mild and self-limiting, however, severe cases can occur in high risk groups (include infants, young children, the elderly and the immunocompromised persons), resulting in high mortality and morbidity in this group.^[3]^ Determining how to prioritize limited food safety resources across a large number of foods is a big challenge in preventing foodborne illness.^[10]^

The reporting of foodborne and waterborne diseases in the United States began approximately 80 years ago, beginning in 1925, the US Public Health Service (PHS) published summaries of outbreaks of gastrointestinal illness attributed to milk.^[11]^ In 1938, PHS added summaries of outbreaks caused by all foods. These early surveillance efforts led to the enactment of important public health measures (e.g., the Pasteurized Milk Ordinance) that resulted in decreased incidence of enteric diseases, particularly those transmitted by milk and water.^[12]^ The current system of surveillance for outbreaks of foodborne and waterborne diseases began in 1966. In 2001, CDC implemented a web-based outbreak surveillance system, the Electronic Foodborne Outbreak Reporting System (eFORS).^[13]^ Developing surveillance for foodborne outbreaks in the United States has posed a formidable challenge.^[14]^ In United States, state and local public health agencies are the frontline for disease surveillance and response activities.^[15,16]^ A 2010 survey of state foodborne disease capacity identified the need for additional staff to reach full capacity; all respondents reported barriers to investigating foodborne disease outbreaks.^[17]^ However, improved surveillance systems in the United States are detecting more outbreaks that would previously have been missed because they are widely dispersed.^[18]^

Since 2000, China has begun to establish 2 networks for monitoring food contaminants and foodborne diseases (“two nets monitoring”). However, after the promulgation and implementation of the food safety law in 2009, China began to establish a food safety risk monitoring system platform. A large number of network data was collected from this platform. And based on the data, it is found out where the food pollution exists in our country to prevent the outbreak of foodborne disease. Shandong is one of the earliest provinces to participate in “two nets monitoring” and food safety risk monitoring.

The study objective was to describe demographic and epidemiological characteristics of foodborne disease outbreaks reported in Shandong Province, China, 2011–2016. To identify and determine the magnitude of the problem, risk factors, monitoring and surveillance and measures of control. Results of this analysis can help guide efforts to prevent foodborne illness.

## 2 Methods

### 2.1 Data source

Shandong Provincial Center for Disease Control and Prvention (SDCDC) conducts surveillance of foodborne disease outbreaks in Shandong Province and has collected data on foodborne disease outbreaks from municipal and county CDCs through the FDOSS 2011. An outbreak of foodborne disease is defied as the occurrence of 2 or more cases of a similar illness resulting from ingestion of a common food. The information collected for each outbreak includes reporting unit, date, region, settings, exact address, type of food source, causes of events, number of illnesses, hospitalizations and deaths, confirmed etiology, the implicated food vehicle, food categories, settings where food was prepared, the etiology, and contributing factors. Laboratory and clinical guidelines for confirming an etiology are specific to each bacterial, chemical/toxin, parasitic, and viral agent. Suspected etiologies are those that do not meet the confirmation guidelines. The cause of the outbreak is categorized as multiple etiologies if more than one etiologic agent is reported.^[19]^ Data were extracted for all foodborne outbreaks in which the first illness occurred during 2011 to 2016.

### 2.2 Data analysis

Frequencies of outbreaks and outbreak-related illnesses, hospitalizations, and deaths were calculated. We also analyzed settings of outbreaks, implicated foods, etiology, and factors contributing to outbreak occurrence. Implicated foods were categorized using the China Food and Drug Administration's scheme.^[20]^ If implicated food(s) that contained ingredients belonging to more than one category that the food category responsible for illness could not be determined, we classified it as ‘complex’. If the organism was detected in samples from 2 or more ill persons, or in an epidemiologically implicated food(s), we defined an etiology as confirmed for most pathogens. For marine and other toxins, a clinically compatible illness in 2 or more ill persons who ate an implicated food was required to confirmation.^[21]^

The Wilcoxon test was used to compare the median number of ill persons in outbreaks. All data analysis was performed using Epi Info 7 (https://www.cdc.gov/epiinfo/support/downloads.html) and the map was performed using ArcGis 10.2.2.

In China, foodborne infectious diseases are not reported in the FDOSS.

## 3 Results

### 3.1 Number of foodborne disease outbreaks

**Figure 1:**
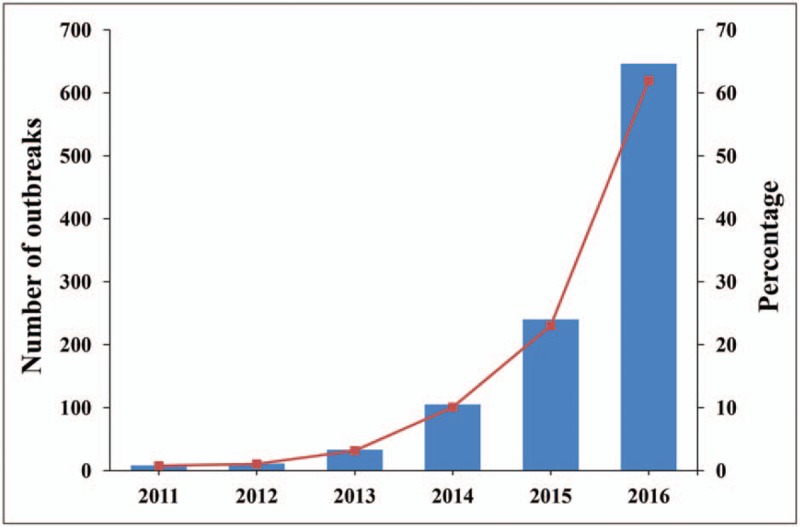
Number of foodborne disease outbreaks and percentage of all foodborne disease outbreaks by year in Shandong Province, Foodborne Disease Outbreak Surveillance System, 2011–2016.

Of 1043 outbreaks reported during 2011 to 2016 in Shandong Province, resulting in 8078 illnesses, 2442 hospitalizations, and 17 deaths. There were a median of 69 outbreaks annually [interquartile range (IQR) 10–342], resulting in 335 to 3824 illnesses each year. The median outbreak size was 3 persons (IQR 2–7). Yantai (232 outbreaks, 22%), Qingdao (158 outbreaks, 15%), and Weihai (86 outbreaks, 8%) reported the largest number of outbreaks. The annual number of outbreaks and the percentage of all outbreaks increased from 2011 (8, 0.8%) to 2016 (431, 61.9%) (Fig. 1)

**Figure 2:**
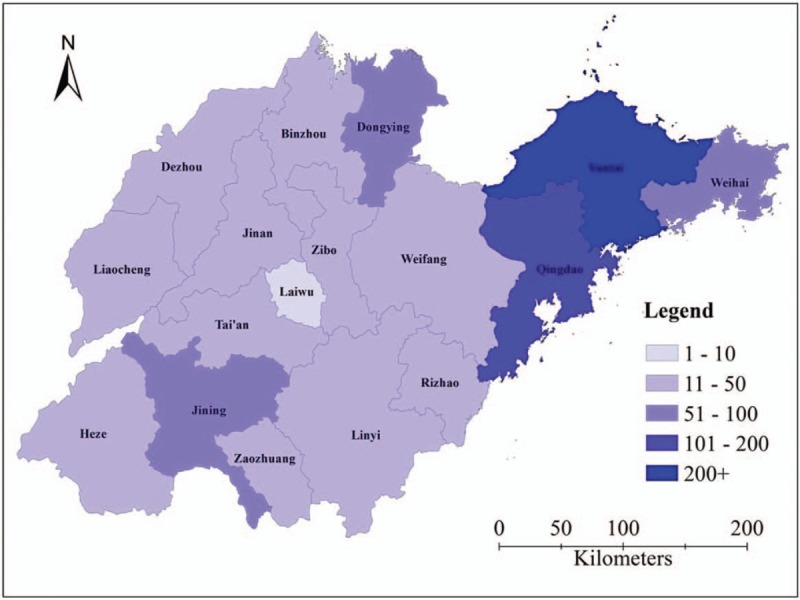
Number of foodborne disease outbreaks by prefecture in Shandong Province, Foodborne Disease Outbreak Surveillance System, 2011–2016.

In Jiaodong peninsula of Shandong Province (including Qingdao,Yantai, and Weihai) reported 483 (46.3%) outbreaks during 2011–2016. Among them, the largest number of reported reports in Yantai (233 outbreaks, 22.3%). The remaining reported outbreaks were distributed in the others 14 prefectures of Shandong Province, no prefecture reported more than 80 outbreaks (Fig. 2).

### 3.2 Settings of outbreaks

In this study, settings of foodborne outbreaks were classified into 14 types. Hotels (including cruise ships, hotpot restaurants, barbecue shops) accounted for 374 (35.9%) outbreaks, resulting in 3237 (40.1%) illnesses, 971 (39.8%) hospitalizations, and 0 deaths. Homes were the second most common setting, accounting for 358 (34.3%) outbreaks, resulting in 1164 (14.4%) illnesses, 501 (20.5%) hospitalizations, and 9 deaths (Table 1).

**Table 1:**
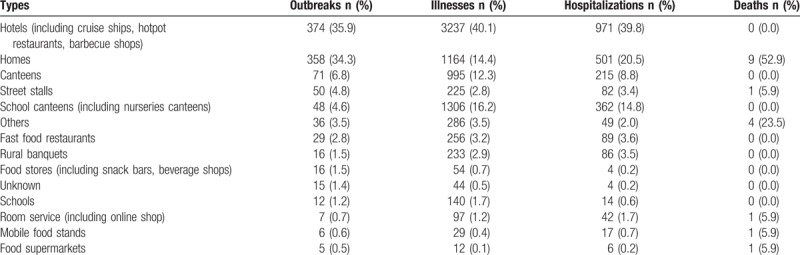
Site types implicated in foodborne disease outbreaks in Shandong Province, Foodborne Disease Outbreak Surveillance System, 2011–2016 (n = 1043).

### 3.3 Implicated foods

Implicated foods were classified into 5 classifications. Of these, 704 (67.5%) had a food that could be assigned to a single food category, complex (216 outbreaks, 20.7%), meat and meat products (138 outbreaks, 13.2%), vegetables (94 outbreaks, 9.0%) were the most common food categories (Table 2).

**Table 2:**
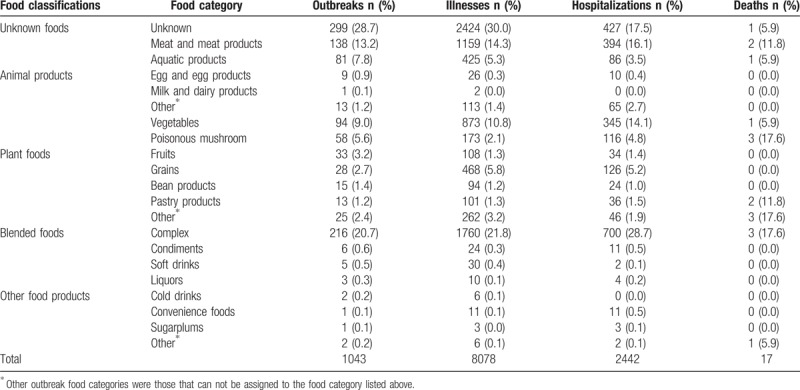
Foods implicated in foodborne disease outbreaks in Shandong Province, Foodborne Disease Outbreak Surveillance System, 2011–2016 (n = 1043).

### 3.4 Etiology

Most outbreaks (747, 71.6%) were not detected out causative agents. In 280 (26.9%) outbreaks, a single confirmed etiology was reported at least (Table 3). The most common etiology was poisonous plants and animals and their toxins, it caused 117 (11.2%) outbreaks. The second was nitrite (39, 3.7%). In poisonous plants and animals and their toxins outbreaks, the median number of ill persons was 3 (IQR 2–6). The median number of ill persons in nitrite outbreaks was 4 (IQR 2–7). Poisonous plants and animals and their toxins caused most deaths (5/17 deaths with a reported etiology, 29.4%).

**Table 3:**
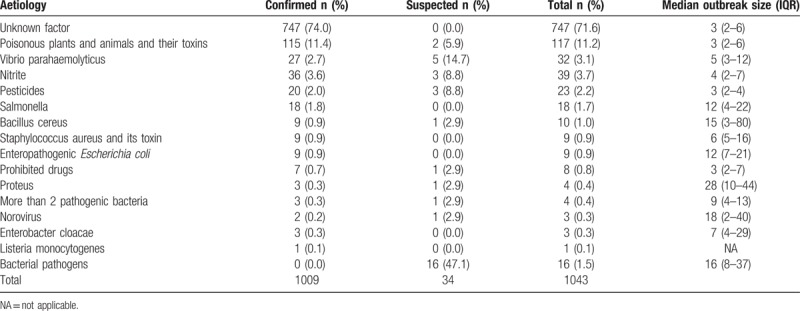
Etiologies in foodborne disease outbreaks in Shandong Province, Foodborne Disease Outbreak Surveillance System, 2011–2016.

Other common confirmed etiologies were vibrio parahaemolyticus (27 outbreaks, 2.7%), pesticides (20 outbreaks, 2.0%) and salmonella (18 outbreaks, 1.8%) (Table 3). The most common pairs in the 280 outbreaks with a confirmed etiology at least linked to a food classification were poisonous plants and animals and their toxins in edible fungi (57 outbreaks, 20.4%) and vegetables (44 outbreaks, 15.7%) of plant foods, nitrite in meat and meat products (19 outbreaks, 6.8%) and vibrio parahaemolyticus in aquatic products (27 outbreaks, 9.6%) of animal products.

### 3.5 Outbreak contributing factors

According to China Foodborne Disease Outbreak Surveillance System, the factors that cause events are divided into: pollution or deterioration of raw materials (auxiliary materials), improper processing, improper storage, inedible and misuse, pesticide residue, illicit use (illicit drugs and additives), pollution of the environment (air, water, soil, etc.), additives abuse and illicit addition, product expired (deterioration), human contamination, pollution of equipments (operating equipment, utensils, etc.), the cause was unknown or not be found out, poisoning and other.

Unknown or unfound cause was the most common contributing factor. It accounted for more than half outbreaks (552, 52.9%), resulting in nearly 40% illnesses, 746 (30.5%) hospitalizations, and 4 (23.5%) deaths. A close second, was improper processing, causing 168 (16.1%) outbreaks, resulting in 2239 (27.7%) illnesses, 701 (28.7%) hospitalizations, and 4 (23.5%) deaths (Table 4).

**Table 4:**
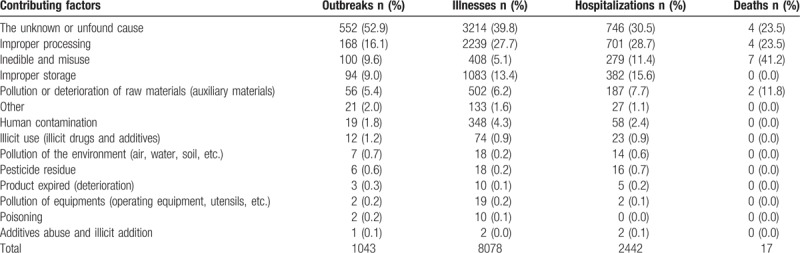
Contributing factors implicated in foodborne disease outbreaks in Shandong Province, Foodborne Disease Outbreak Surveillance System, 2011–2016 (n = 1043).

## 4 Discussion

Data collected during outbreak investigations provide insights into the pathogens and foods that cause illness although relatively few of these illnesses occur in the setting of a recognized outbreak.^[1]^ Previous research, however, suggests that socioeconomic status plays an important role in the causation of foodborne illnesses. Populations with low socioeconomic status have less access to high-quality food products, resulting in reliance on small markets that may sell foods of poorer quality. However, our analysis does not support the above view, this may be related to the fact that many foodborne cases reported in the Jiaodong Peninsula are not local cases, because Jiaodong peninsula is an economically developed area in Shandong province, and it is also a developed tourist area, a large number of foreign tourists travel to this area every year, it accounted for nearly half of foodborne disease outbreaks in Shandong province during 2011 to 2016.

Nearly 36% of all foodborne disease outbreaks reported to SDCDC from 2011 to 2016 involved in a hotel setting. This likely indicates foodborne disease outbreaks involved foods prepared most often in commercial settings.^[22,23]^ Over half of all foodborne disease outbreaks had a food that could be assigned to a single food category. It means that most of the implicated foods were contaminated at least one link in the process of processing and consumption.

In the identified outbreaks, we found that factors related to improper processing foods (including handling, preparation practices, et al) were the most frequent contributors to outbreaks. Time pressure, sink accessibility and clean cutting board availability, structural environments, equipment, and resources, management and coworker influence, worker characteristics, lack of food safety education and training and procedures have been reported as obstacles of food workers to impact their ability to safely prepare foods in accordance with guidelines and regulations^[24]^ and are likely reasons for food preparation lapses. In these outbreaks, the most common confirmed etiology was poisonous plants and animals and their toxins. This likely indicates that food materials were misused or inadequate cooking.

Particular attention should be paid to toxic mushroom poisoning. Mushroom has been a source of diet and article of commerce since long time and across many cultures in China. An estimate of more than 1000 kinds of poisonous mushrooms in nature, while in China at least 500 kinds.^[25,26]^ Unintentional consumption of poisonous wild mushrooms resulting in poisoning. In Shandong Province, poisonous mushroom poisoning is one of the leading causes of death.

The likelihood that public health authorities are alerted about an outbreak depends on many factors, including its size and the severity of illnesses; consumer and physician awareness, interest, and motivation to report the incident; and the resources and disease surveillance activities of state and local public health and environmental agencies.^[27]^ Besides, the investigation and treatment of foodborne disease outbreaks is led by FDA in China, and CDC is only responsible for the field epidemiological investigation. CDC can only carry out investigation after receiving notification from the higher authorities or FDA. These may explain why most outbreaks were not detected out causative agents and the cause was unknown or not be found out was the most common contributing factor, but it is consistent with reports in the United States.^[28]^ Capacity of municipal and county CDCs to investigate foodborne disease outbreaks varies widely, with the most notable limitations being lack of dedicated personnel and delayed notification of outbreaks.^[29]^ Efforts to better understand the gaps in foodborne outbreak response, including laboratory, epidemiologic, and environmental health capacity, may ultimately inform strategies to overcome the challenges of limited public health resources.^[30]^ Globally, due to limitations of laboratory capacities in diagnosis of etiology, most of foodborne outbreak agents remain unknown.^[31,32]^

## 5 Limitations of this study

We know the fact that the quality and quantity of investigations reported determine the quality of outbreak data. A limitation of our study is that a lack of information on certain aspects of the outbreak (e.g., the etiology or the implicated food vehicle) is missing or incomplete for many reports, conclusions drawn from confirmed outbreaks may not apply to all outbreaks. Outbreaks in some settings (e.g., families and food supermarkets) are not easily to be recognized and investigated is also a limitation, because the settings in which contaminated food is prepared and consumed might not fully reflected by data on the places where outbreaks occurred. In addition, some outbreaks were investigated and disposed, but for various reasons, they were not reported through the system; and even some outbreaks or smaller outbreaks might not be investigated or reported to local CDC or health departments are limitations, too. Therefore, the results of this analysis represent data available at a single point in time and might differ from those published earlier or later.

## 6 Conclusions

This study summarizes the most recently reported cases of food-borne disease outbreaks in Shandong Province. To analyze foodborne disease outbreak investigations reported by local CDC help to understand the epidemiology of foodborne disease in Shandong Province. These findings underline the importance of targeted prevention measures for the specific settings and foods that are associated with the most outbreaks and illnesses (i.e., hotels, homes, animal products, and plant foods). Only a very few foodborne diseases can not be prevented, so timely investigation, disposal and reporting of foodborne disease outbreaks provides information that might help to reduce foodborne illnesses. Departments responsible for foodborne diseases can use these data to help target efforts to prevent contamination of foods from farm to consumption. China Foodborne Disease Outbreak Surveillance System in Shandong Province should be continued and strengthened even more, such as an increase in diagnostic laboratory capacities. Continued surveillance for foodborne disease outbreaks is important to understand changes in the foods, settings, and pathogens associated with illness.

## Author contributions

**Conceptualization:** Ya Liu, Zhongjun Du.

**Data curation:** Guangjian Wu, Qun Yuan, Liansen Wang, Maoqiang Zhuang, Ya Liu, Zhongjun Du.

**Funding acquisition:** Zhongjun Du.

**Methodology:** Guangjian Wu, Liansen Wang, Jinshan Zhao, Zunhua Chu, Yingxiu Zhang, Kebo Wang, Peirui Xiao, Zhongjun Du.

**Project administration:** Guangjian Wu, Liansen Wang, Maoqiang Zhuang, Kebo Wang.

**Software:** Qun Yuan, Jinshan Zhao, Zunhua Chu, Peirui Xiao.

**Writing – original draft:** Guangjian Wu.

**Writing – review & editing:** Yingxiu Zhang, Ya Liu, Zhongjun Du.

Zhongjun Du orcid: 0000-0001-7422-6035.
